# Using the Health Belief Model to Understand Age Differences in Perceptions and Responses to the COVID-19 Pandemic

**DOI:** 10.3389/fpsyg.2021.609893

**Published:** 2021-04-15

**Authors:** Lauren E. Bechard, Maximilian Bergelt, Bobby Neudorf, Tamara C. DeSouza, Laura E. Middleton

**Affiliations:** Department of Kinesiology, University of Waterloo, Waterloo, ON, Canada

**Keywords:** COVID-19, health belief model, health behavior, health communication, aging, public health practice

## Abstract

COVID-19 severity and mortality risk are greater for older adults whereas economic impact is deeper for younger adults. Using the Health Belief Model (HBM) as a framework, this study used a web-based survey to examine how perceived COVID-19 susceptibility and severity and perceived efficacy of recommended health behaviors varied by age group and were related to the adoption of health behaviors. Proportional odds logistic regression was used to examine the relationship between age group and perceived COVID-19 susceptibility, severity, impact, and health behavior efficacy and adoption. Structural equation modeling based on HBM constructs examined the relationships between health beliefs and behaviors. Data from 820 participants (Ontario, Canada) were analyzed (age: 42.7, 16.2 years; 79% women). Middle-aged and older adults reported greater concerns about the personal risk of hospitalization and mortality, economic impact, and social impact of COVID-19 than young adults. Middle-aged adults also reported greatest concern for other age groups. Adoption and perceived efficacy of health behaviors was similar across age groups with few exceptions. Both middle-aged and older-adults were more likely to perceive their own and each other's age groups as responding adequately to COVID-19 compared to young adults. Structural equation modeling indicated perceived benefits of health behaviors were the primary driver of behavior uptake, with socioeconomic factors and perceived severity and susceptibility indirectly associated with uptake through their influence on perceived benefits. Overall, these results suggest adoption of health behaviors is very high with few differences between age groups, despite differences in perceived impact of COVID-19. Public health communications should focus on the benefits of health behaviors to drive adoption.

## Introduction

In December 2019, a cluster of pneumonia cases of unknown origin was reported in Wuhan, China (Bogoch et al., 2020). The novel coronavirus SARS-COV-2, more commonly known as COVID-19, was later determined to be the cause (Jiang et al., 2020). The World Health Organization (WHO) declared COVID-19 a global pandemic on March 11, 2020 (World Health Organization, 2020). As of March 4, 2021, there were over 115 M global confirmed cases of COVID-19 and over 2.5 M deaths (Johns Hopkins University of Medicine, 2021), with a case fatality rate estimated to be 0.25–3.0% (Wilson et al., 2020). While all age groups are susceptible to COVID-19 infection, older age groups have increased risk of severe symptoms and mortality (Onder et al., 2020; Ruan et al., 2020; Zhou et al., 2020). Case fatality among adults over 65 years is approximately 4 times that of young adults (Guo et al., 2020).

Until herd immunity are achieved, containment of the COVID-19 pandemic continues to rely on reduction of exposure through personal health behaviors (e.g., physical distancing, hand-washing, avoidance of face touching) and governmental restrictions (e.g., mandatory isolation periods, boarder closures) (Wilder-Smith and Freedman, 2020). Adherence to public health measures can limit the rate of transmission, thus reducing the number of cases and fatalities and the risk of overwhelming health systems (Jarvis et al., 2020; Tuite et al., 2020). In the first 3-months of the COVID-19 pandemic, rates of COVID-19 and mortality across countries were highly dependant on the extent and timing of public health measures (Ng et al., 2020; Pan et al., 2020; Rocklöv et al., 2020). Analysis of the public health measures in relation to COVID-19 incidence estimated that over 50 million cases were prevented in the first 26 days of the pandemic by implementation of public health measures in China, South Korea, Iran, Italy, France, and the United States alone (Hsiang et al., 2020).

While public health restrictions are critical to containing the spread of COVID-19, infection prevention and control measures have had substantial repercussions for personal economic, physical, social, and mental well-being, which are likely to persist beyond the COVID-19 pandemic. Unemployment in the Canada has risen over the course of the pandemic from 5.6% in January 2020 pre-pandemic to 9.4% in January 2021 (Statistics Canada, 2021). The number of people seeking care for other serious health conditions has decreased substantially (e.g., stroke, heart attacks, cancer), which will likely exacerbate management of these health conditions in the long-term (de Pelsemaeker et al., 2020; De Rosa et al., 2020; Dinmohamed et al., 2020; Pessoa-Amorim et al., 2020). Social distancing and quarantine recommendations have disrupted important social connections, negatively impacting mental well-being by increasing levels of loneliness and psychological distress as observed following other virus outbreaks (Hawryluck et al., 2004; Jeong et al., 2016; Brooks et al., 2020; Williams et al., 2020). For example, evidence indicates increased illicit drug-related overdoses during the COVID-19 pandemic (American Medical Association, 2020; Canadian Centre on Sunbtance Use Addiction, 2020).

The type and severity of COVID-19's impacts vary across age groups. While the immediate health impact of COVID-19 infection is more severe among older adults, the economic impact of COVID-19 can be greater for young adults (Popplewell, 2020). Loss of income was reported by 80% of young adults during the first wave of COVID-19, but only two-thirds of middle-aged adults and less than half of older adults (who are retired more often) reported loss of income during this same time period (Popplewell, 2020). Furthermore, young adults are employed more often in sectors that have seen greater job loss due to COVID-19 disruptions (e.g., service sector, gig economy) (Bell and Blanchflower, 2020).

The Health Belief Model (HBM) is an empirically-supported model of health behavior that provides a framework for understanding how the adoption of public health measures is driven by perceptions of COVID-19 risk and the benefits and barriers to recommended health behaviors for reducing COVID-19 transmission (Rosenstock, 1974; Janz and Becker, 1984; Champion and Skinner, 2008). The HBM consists of six core constructs: perceived severity and susceptibility of the condition, perceived benefits and barriers to the recommended health behavior, and cues to action (immediate prompts for the behavior) and self-efficacy to uptake behavior (Champion and Skinner, 2008). The HBM also considers factors that can moderate the relationships among key constructs (e.g., age group) (Jones et al., 2014). The HBM was originally developed to understand acceptance of preventive measures or screening for early detection of asymptomatic disease (Rosenstock, 1974) but has been used to study the uptake of and compliance to a range of health behaviors. The HBM has been used to specifically study the uptake of health behaviors recommended to avoid seasonal influenza in elderly adults and to characterize perceptions of physical distancing for COVID-19 prevention (Kan and Zhang, 2018; Raamkumar et al., 2020).

Since the health, social, and economic impacts of COVID-19 varies by age group, the cost-to-benefit trade-offs of recommended health behaviors may be perceived as more severe among younger than older adults. An understanding of decision making related to the adoption of these health behaviors is imperative for managing the continuing COVID-19 pandemic response. As a result, the objectives of this study were to: (1) compare perceived COVID-19 susceptibility, severity, and personal, social, and economic impacts across age groups; (2) compare the perceived efficacy of COVID-19 infection control health behaviors amongst different age groups; (3) apply the HBM framework to understand the relationships between health beliefs and behaviors for preventing COVID-19 infection and transmission; (4) describe perceptions of how well various age groups are responding to the COVID-19 pandemic.

## Materials and Methods

### Study Design

This was a cross-sectional survey conducted from May 7, 2020 to June 5, 2020. Adults across all age groups were enrolled through convenience sampling. Participants were recruited by circulating a weblink to the survey using social media (Twitter, Facebook, LinkedIn) and email listservs at the authors' research institution. Secondary sharing of recruitment materials by word of mouth within personal networks of survey respondents supported broader dissemination of the survey. This study was reviewed and approved by the University of Waterloo Office of Research Ethics (ORE#42131). All participants provided informed consent by choosing “yes” after reviewing study information, acknowledging that they understood study information and potential risks.

### Survey

A survey was developed by the research team to collect sociodemographic, perceived health and COVID-19-related health impacts, beliefs, and behaviors was delivered using Qualtrics (Qualtrics, Provo, UT). The full survey is provided in Supplementary File 1. This survey was informed by the domains of the HBM (as well as theoretically confounding variables). Where possible, demographic questions and format of COVID-19 questions was based on Statistics Canada survey question. Where we did not identify any directly relevant survey questions, wording and responses were based on recommended Likert scale wording for similar questions. Although there was no formal validity or reliability evaluation, the survey was piloted among 6 local researchers not involved in the study to provide feedback on usability and face validity of questions prior to dissemination.

#### Demographic and Health-Related Characteristics

Participants reported demographics including age, gender, ethnicity, highest level of education, income, employment status, and country, province/state and city of residence. Using location data, participants were classified as residing in or not residing in large urban centers (≥100,000 people) based on the most recent census population data (Raamkumar et al., 2020) or a web search of population for regions that were not included in Canadian census data. Participants also reported whether their occupation was health-related and, if so, their role (health care provider, health care administrator, public health professional, community health worker, health researcher, or other). Participants described self-rated physical and mental health and need for assistance with everyday activities. Participants also reported whether they: (1) had tested positive for COVID-19; (2) whether they suspected that they had COVID-19; (3) whether they suspected they had been exposed to COVID-19; and (4) whether they had a close family member or friend who had COVID-19.

#### Health Behaviors and Perceived Effectiveness

Participants reported frequency of adhering to recommended COVID-19 health behaviors. These included: (1) avoiding leaving home for non-essential reasons; (2) using social distancing when out in public; (3) avoiding crowds and large gatherings; (4) washing hands more frequently; (5) avoiding touching their face; (6) working from home; and (7) canceling non-essential travel; and (8) other (specified). Participants were also able to specify other health behaviors. Of note, wearing a face mask was not yet widely recommended at the start of the survey, so it was not specifically listed as a health behavior; however, participants could choose to report masks in the “other” category. Frequency of adherence to and perceived effectiveness of the health behaviors was reported using a 5-point Likert scale (never, rarely, sometimes, very often, always).

#### Perceived Severity and Susceptibility of COVID-19

Participants reported their level of concern about the impact of COVID-19 for their personal health, including being infected themselves, being hospitalized if infected, and dying if infected along a scale of five categories of increasing concern (not at all concerned, somewhat concerned, moderately concerned, very concerned, extremely concerned). Participants also reported their level of concern about the impact of COVID-19 on their social and financial well-being. These concerns included: ability to meet their financial obligations, their economic future, and maintaining social connections. Participants were also able to specify another impact that was not listed for each type of impact. All concerns were reported using the same 5-point Likert scale, as above.

#### Perceived Threat of COVID-19 to Others by Age Group

Participants reported their level of concern (using the 5-point Likert scale described above) about the impacts of COVID-19 to the health, economic future, and social well-being of youth (<18 years), young adults (18–34 years), middle-aged adults (35–64 years), and older adults (≥65 years).

#### Perception of Others' Health Behaviors

Participants reported whether youth (under 18 years old), young adults (18–39 years old), middle-aged adults (40–64 years old), and older adults (65 years and older) were doing enough to reduce the spread of COVID-19. Participants responded using a 5-point Likert scale (definitely not, probably not, might or might not be, probably yes, definitely yes).

### Statistical Analysis

Of 1,105 submitted surveys, we restricted our analyses to complete surveys where participants were at least 18 years old and took at least a minute to complete the survey. Additionally, due to low response rates from other regions, we restricted our analyses to respondents who reported their province of residence as Ontario, Canada, leaving us with 820 participants. For analyses that controlled for gender, only those who answered male or female were included (*n* = 813). Missing data ranged from 0 to 6.2% across variables of interest, with an average of 1%. missRanger v2.1.0 package was used to impute data (Mayer, 2019). This package imputes data by chaining random forests in order to predict missing values based on the missForest package. Between the iterative model fitting it performs, this method also uses predictive mean matching to avoid imputation of values not present within the original data, and raises the variance in the resulting conditional distributions to more realistic levels. The random forest algorithm works well for datasets with mixed type data and accounts for complex interactions and non-linear relations. Conceptually, this is similar to the expectation maximization procedure in that it seeks to predict the most plausible value for missing items from other variables in the dataset. To simplify analyses, we used a single imputed dataset (i.e., we did not perform multiple imputation) since the missing data rate was very low so the effect of single imputation vs. multiple imputation was likely to be negligible.

All analyses were performed using R v4.0.1. Impact of age group (young adult, 18–39 years; middle-aged adult, 40–64 years; and older adult, ≥65 years) on outcome variables was analyzed using partial proportional odds logistic regression [clm() function of the ordinal package v2019.12-10] (Christensen, 2019). Very few participants reported low frequency of adherence to health behaviors, resulting in a clustering of responses and unstable models. As a result, we collapsed the bottom 3 response categories for health behavior frequency (“Never,” “Rarely,” “Sometimes”) into a single category and maintained the other two response options separately (“Very Often,” “Always”). We regressed the outcome variables on age group (“young adult,” 18–39 years; “middle-aged adult,” 40–64 years; “older adult,” ≥65 years), our predictor of interest. We also included variables that were likely to influence health behaviors based on prior research but were not included in the HBM, including gender, ethnicity (Caucasian/other), residence in large urban center (yes/no), household income, employment status (employed vs. unemployed), self-reported physical health, self-reported mental health, education (post-secondary vs. no post-secondary), health-related occupation (yes/no) and exposure to COVID-19 (whether they had perceived self-exposure, a family member diagnosed, suspected that they had had COVID-19, or themselves were diagnosed with COVID-19). The proportional odds assumption for variables in the model was tested using the nominal_test function from the ordinal package. For the control variables, if the nominal_test indicated a violation of the proportional odds assumption (for this purpose a *p*-value of <0.1) we specified the variable as having a nominal effect, resulting in a partial proportional odds model. Some models experienced convergence difficulties when run with some of the control variables and so those control variables had to be dropped. Where this happened, we have indicated what variables had to be dropped. We made no changes to the specification of the age group variable based on results of the nominal_test; we provide all results along with the nominal_test *p*-value for this variable. Younger adults were the reference group in all analyses, as increased age was considered the added exposure for risk of COVID-19 severity.

Structural equation modeling was used to assess the relationship between perceived health benefits, perceived personal severity and susceptibility, socioeconomic barriers, and health behaviors using the sem() function from the lavaan package v0.6-6 (Rosseel, 2012). The model was theoretically defined before examining the data. The model was registered online prior to analysis^1^. The *Perceived Health Benefits* latent variable was comprised of survey items corresponding to belief in the efficacy of 5 health behaviors: social distancing, staying home, avoiding crowds, canceling travel, and working from home. The *Reported Health Behaviors* latent variable was comprised of survey items reporting the frequency in which participants engaged in these same 5 health behaviors. The behaviors “washing hands” and “avoiding touching face” were not included as these were health behaviors which we did not believe to be related to socioeconomic barriers. The *Socioeconomic Barriers* latent variable was made up of participants' self reported concerns for their financial future, their financial obligation, their social connections, and their self reported income bracket (5 categories, higher numeric values correspond to lower incomes). The *Perceived Personal Severity and Susceptibility* latent variable was comprised of survey items corresponding to self-reported concerns of being infected, being hospitalized, dying, or other concerns defined by participants.

To assess the relationship between latent variables, the *Health Behavior* latent variable was regressed on the *Socioeconomic Barriers, Perceived Personal Severity and Susceptibility*, and *Perceived Health Benefits* latent variables. The *Socioeconomic Barriers, Perceived Personal Severity and Susceptibility*, and *Perceived Health Benefits* latent variables were allowed to correlate with each other. Latent variables were scaled by fixing a reference indicator, the lavaan default. To address issues of non-normality, a robust version of the maximum likelihood estimator was used (MLM). For our purposes, a “good” model fit is indicated by SRMR close to.08, RMSEA close to.06, and CFI close to 0.95 (Hu and Bentler, 1999).

## Results

### Study Sample

The average age of the study sample was 42.7 years (SD = 16.2 years), with an age range of 18–83 years. The study sample was predominantly women (79.3%) and Caucasian (81.4%), and most had at least some post-secondary education (94.7%). Almost a quarter of the study sample (24.0%) worked in health-related occupations. There were significant differences between age groups in terms of employment status and occupation, with more older adults being retired. Almost a quarter of the sample believed they had been exposed to COVID-19 (*n* = 189, 23.3%), and more people believed they had been infected with COVID-19 (*n* = 93, 11.5%) than reported receiving positive test result for COVID-19 (*n* = 2, 0.2%). More older adults believed they had been exposed to COVID-19 at the time of the survey. Detailed participant characteristics by age group are provided in Table 1.

**Table 1:** Participant characteristics by age group [*n* = 820; mean (sd) or % (*n*)].

**Characteristics**	**Total**	**Young adults**	**Middle-aged adults**	**Older adults**	***P*-value**
Age	42.7 (16.2)	28.2 (6.2)	52.9 (7.0)	70.2 (4.6)	<0.0001
Gender, women	79.3% (645)	78.3% (306)	81.9% (281)	73.4% (58)	0.1904
Education, at least some post-secondary education	94.7% (769)	94.9% (371)	95.9% (328)	88.6% (70)	0.0265
Ethnicity, Caucasian	81.4% (633)	72.4% (275)	89.8% (289)	90.8% (69)	0.0005
Employment status					0.0005
Employed	61.1% (495)	67.6% (263)	66.7% (228)	5.1% (4)	
Not employed	18.0% (146)	28.5% (111)	9.9% (34)	1.3% (1)	
Self-employed	7.7% (62)	3.9% (15)	11.1% (38)	11.4% (9)	
Retired	13.2% (107)	0.0% (0)	12.3% (42)	82.3% (65)	
Large-urban city, yes	86.8% (686)	90.0% (342)	83.7% (278)	84.6% (66)	0.0380
Health-related occupation	24.0% (192)	28.9% (112)	22.3% (75)	6.8% (5)	0.0005
COVID-19 exposure (% at least 1)	34.2% (278)	37.4% (146)	34.4% (118)	17.7 % (14)	0.0035
Believe exposed to COVID-19	23.3% (189)	27.4% (107)	22.1% (75)	8.9% (7)	0.0015
Believe infected with COVID-19	11.5% (93)	11.5% (45)	13.2% (45)	3.8% (3)	0.0500
Tested positive for COVID-19	0.2% (2)	0.0% (0)	0.6% (2)	0.0% (0)	0.3683
Family member had COVID-19	12.7% (103)	11.8% (46)	14.6% (50)	8.9% (7)	0.3008
Income, < $50,000	19.1% (150)	26.5% (101)	9.6% (32)	24.6% (17)	0.0005
Need help with everyday activities, Yes	1.5% (12)	1.8% (7)	1.5% (5)	0.0% (0)	0.5032
Self-rated physical health, /100	78.6 (14.9)	77.6 (15.9)	79.5 (14.4)	79.5 (11.6)	0.2011
Self-rated mental health, /100	74.2 (20.1)	68.5 (21.2)	78.6 (17.9)	82.5 (16.3)	<0.0001

*Young adult = 18–34 years; Middle-aged adults = 35–64 years; Older adults = 65+ years*.

### Health Beliefs About COVID-19

Table 2 shows perceived severity of the impact of COVID-19 to one's economic well-being, social well-being, and personal health. In brief, compared to young adults, middle-aged and older adults had greater concerns about COVID-19 infection (OR 2.85, 95% CI 1.96–4.16 and OR 2.30, 95% CI 1.26–4.16, respectively), hospitalization (OR 3.12, 95% CI 2.25–4.36 and OR 4.08, 95% CI 2.41–6.93, respectively), and death (OR 2.82, 95% CI 1.98–4.05 and OR 3.65, 95% CI 2.08–6.43, respectively). Older adult respondents were less likely to be concerned about meeting current financial obligations than were younger adults (OR 0.30, 95% CI 0.11–0.70) and less likely to be concerned about the impact of COVID-19 on their economic future (OR 0.52, 95% CI 0.27–0.97). Middle-aged adults were marginally more concerned (OR 1.43, 95% CI 0.99–2.08) about the impact of COVID-19 on their ability to maintain their social connections and older adults were significantly more concerned about this (OR 2.22, 95% CI 1.20–4.05).

**Table 2:** Level of concerns about personal impact of COVID-19 infection [% (*n*)].

**Age group**	**Up to moderately concerned**	**Very concerned**	**Extremely concerned**	**Odds ratio (95% CI)**	***P*-value**	**PO assumption**
**IMPACT TO PERSONAL HEALTH**						
**Being infected with COVID-19**						
Young adults (ref)	80% (314)	15% (58)	5% (19)	1.0	–	0.9225
Middle-aged adults	63% (217)	24% (84)	12% (42)	2.85 (1.96-4.16)	<0.0001	
Older adults	63% (50)	25% (20)	11% (9)	2.30 (1.26-4.16)	0.0065	
**Being hospitalized if infected**						
Young adults (ref)	67% (263)	20% (79)	13% (49)	1.0	–	0.0527
Middle-aged adults	43% (149)	35% (121)	21% (73)	3.12 (2.25-4.36)	<0.0001	
Older adults	33% (26)	42% (33)	25% (20)	4.08 (2.41-6.93)	<0.0001	
**Dying of COVID-19**						
Young adults (ref)	77% (301)	11% (43)	12% (47)	1.0	–	0.3891
Middle-aged adults	58% (200)	21% (72)	21% (71)	2.82 (1.98-4.05)	<0.0001	
Older adults	51% (40)	23% (18)	27% (21)	3.65 (2.08-6.43)	<0.0001	
**PERCEIVED PERSONAL IMPACT OF COVID-19**						
**Meet financial obligations**						
Young adults (ref)	76% (298)	15% (57)	9% (39)	1.0	–	0.9784
Middle-aged adults	80% (274)	13% (45)	7% (24)	1.44 (0.96-2.16)	0.0805	
Older adults	92% (73)	5% (4)	3% (2)	0.30 (0.11-0.70)	0.0102	
**Economic future**						
Young adults (ref)	65% (253)	23% (90)	12% (48)	1.0	–	0.5350
Middle-aged adults	69% (238)	20% (70)	10% (35)	1.25 (0.89-1.77)	0.2052	
Older adults	77% (61)	19% (15)	4% (3)	0.52 (0.27-0.97)	0.0446	
**Maintaining social connections**						
Young adults (ref)	76% (297)	18% (69)	6% (25)	1.0	–	0.5559
Middle-aged adults	74% (255)	16% (56)	9% (32)	1.43 (0.99-2.08)	0.0577	
Older adults	66% (52)	27% (21)	8% (6)	2.22 (1.20-4.05)	0.0101	

*Young adult as reference, OR = 1.0; Young adults = 18–34 years; Middle-aged adults = 35–64 years; Older adults = 65+ years*.

Level of concern amongst age groups about the impact of COVID-19 on personal health, economic future, and social well-being for one's own and other age groups are reported in Table 3. Middle-aged and older adults were more likely to report greater concern for the health impacts of COVID-19 for youth and young adults relative to young adults. Middle-aged adults were also more likely to report greater concern for other middle-aged adults. When it came to the health impact on older adults, middle-aged adults were marginally more concerned while older adults were marginally less concerned. Middle-aged and older adults also reported higher concern for the economic futures of youth, middle-aged adults, and older adults compared to young adults. Concerns about social impacts were more similarly rated across age groups, with the only differences being that middle-aged adults were more likely to report higher concern for older adults (1.56, 1.16–2.11) and older adults were more likely to report higher concern for youth (1.87, 1.12–3.13) than did young adults.

**Table 3:** Level of concern about impact of COVID-19 on health, economic, and social well-being of age groups.

**Age group**	**Up to moderately concerned**	**Very concerned**	**Extremely concerned**	**Odds ratio (95% CI)**	***P*-value**	**PO assumption**
**IMPACT ON PERSONAL HEALTH**						
**Impact in youth**						
Young adults (ref)	81% (318)	13% (49)	6% (24)	1.0	–	0.5705
Middle-aged adults	67% (229)	23% (79)	10% (35)	2.77 (1.89-4.10)	<0.0001	
Older adults	59% (47)	28% (22)	13% (10)	3.02 (1.66-5.45)	0.0003	
**Impact in young adults**						
Young adults (ref)	80% (313)	17% (66)	3% (12)	1.0	–	0.1667
Middle-aged adults	62% (214)	28% (96)	10% (33)	3.08 (2.13-4.51)	<0.0001	
Older adults	59% (47)	35% (28)	5% (4)	2.02 (1.12-3.60)	0.0177	
**Impact in middle age**						
Young adults (ref)	46% (181)	40% (155)	14% (55)	1.0	–	0.2811
Middle-aged adults	42% (143)	39% (134)	19% (66)	1.48 (1.09-2.01)	0.0123	
Older adults	38% (30)	48% (38)	14% (11)	1.15 (0.69-1.92)	0.5866	
**Impact in older adults**						
Young adults (ref)	4% (17)	34% (134)	61% (240)	1.0	–	0.063
Middle-aged adults	6% (21)	25% (85)	69% (237)	1.40 (1.00-1.96)	0.0524	
Older adults	11% (9)	35% (28)	53% (42)	0.58 (0.34-1.02)	0.0588	
**IMPACT TO ECONOMIC FUTURE**						
**Impact in youth**						
Young adults (ref)	74% (288)	17% (66)	9% (37)	1.0	–	0.5421
Middle-aged adults	57% (194)	27% (94)	16% (55)	2.42 (1.72-3.40)	<0.0001	
Older adults	48% (48)	27% (21)	25% (20)	3.38 (1.96-5.82)	0	
**Impact in young adults**						
Young adults (ref)	33% (128)	40% (158)	27% (105)	1.0	–	0.8748
Middle-aged adults	38% (130)	40% (138)	22% (75)	0.93 (0.69-1.25)	0.6209	
Older adults	30% (24)	44% (35)	25% (20)	1.06 (0.64-1.76)	0.8320	
**Impact in middle age**						
Young adults (ref)	56% (218)	33% (130)	11% (43)	1.0	–	0.4248
Middle-aged adults	37% (127)	41% (140)	22% (76)	2.35 (1.73-3.21)	<0.0001	
Older adults	29% (23)	49% (39)	22% (17)	2.45 (1.47-4.11)	0.0006	
**Impact in older adults**						
Young adults (ref)	71% (279)	19% (76)	9% (36)	1.0	–	0.5132
Middle-aged adults	54% (186)	27% (91)	19% (66)	2.44 (1.75-3.41)	<0.0001	
Older adults	54% (43)	32% (25)	14% (11)	2.89 (1.66-5.04)	0.0002	
**Social wellbeing**						
**Impact in youth**						
Young adults (ref)	46% (180)	34% (132)	20% (79)	1.0	–	0.3964
Middle-aged adults	46% (157)	31% (106)	23% (80)	1.17 (0.86-1.58)	0.3202	
Older adults	47% (29)	38% (30)	25% (20)	1.87 (1.12-3.13)	0.0164	
**Impact in young adults**						
Young adults (ref)	54% (211)	29% (114)	17% (66)	1.0	–	0.8139
Middle-aged adults	53% (181)	31% (107)	16% (55)	1.25 (0.92-1.71)	0.1544	
Older adults	47% (37)	35% (28)	18% (14)	1.64 (0.97-2.78)	0.0659	
Impact in middle age^*^						
Young adults (ref)	63% (245)	24% (93)	14% (53)	1.0	–	0.6262
Middle-aged adults	60% (207)	28% (96)	12% (40)	1.32 (0.96-1.83)	0.0914	
Older adults	58% (46)	27% (21)	15% (12)	1.49 (0.86-2.57)	0.1559	
**Impact in older adults**						
Young adults (ref)	33% (130)	36% (142)	30% (119)	1.0	–	0.8275
Middle-aged adults	29% (98)	36% (125)	35% (120)	1.56 (1.16-2.11)	0.0035	
Older adults	38% (30)	35% (28)	27% (21)	1.07 (0.64-1.78)	0.7939	

*Young adult as reference, OR = 1.0; Young adults = 18–34 years; Middle-aged adults = 35–64 years; Older adults = 65+ years*.

* *Education control variable dropped due to convergence issues*.

### Adoption and Perceived Effectiveness of Health Behaviors

Survey respondents indicated their likelihood of engaging in specific health behaviors to reduce the risk of COVID-19 infection and transmission and their perceived effectiveness of these behaviors (Table 4). Both middle-aged (OR 0.45, 95% CI 0.31–0.64) and older (OR 0.23, 95% CI 0.13–0.41) adults were less likely to report working from home than young adults. Middle-aged adults were less likely to report staying home (OR 0.72, 95% CI 0.53–0.98) but were more likely to report social distancing (OR 1.61, 95% CI 1.12–2.33), washing hands (OR 1.44, 95% CI 1.04–1.99), and avoiding touching their face (OR 1.43, 95% CI 1.06–1.93) than were young adults.

**Table 4:** Likelihood of reporting health behaviors across age groups.

**Age group**	**Sometimes or less**	**Very often**	**Always**	**Odds ratio (95% CI)**	***P*-value**	**PO assumption**
**LIKELIHOOD OF REPORTING HEALTH BEHAVIORS**						
**Staying home**						
Young adults (ref)	16 (62)	46 (181)	38 (148)	1.0	–	0.8228
Middle-aged adults	20 (67)	50 (173)	30 (103)	0.72 (0.53–0.98)	0.0351	
Older adults	22 (17)	46 (36)	33 (26)	0.79 (0.47–1.34)	0.3810	
**Social distancing**						
Young adults (ref)	3 (12)	28 (111)	69 (268)	1.0	–	0.8379
Middle-aged adults	2 (7)	20 (68)	78 (268)	1.61 (1.12–2.33)	0.0106	
Older adults	3 (2)	16 (13)	81 (64)	1.74 (0.90–3.54)	0.1113	
**Avoiding crowds**						
Young adults (ref)	3 (13)	10 (40)	86 (338)	1.0	–	0.6276
Middle-aged adults	3 (9)	8 (29)	89 (305)	1.34 (0.81–2.23)	0.2532	
Older adults	4 (3)	5 (4)	91 (72)	1.60 (0.65–4.38)	0.3289	
**Washing hands**						
Young adults (ref)	13 (49)	34 (133)	53 (209)	1.0	–	0.2524
Middle-aged adults	6 (20)	31 (107)	63 (216)	1.44 (1.04–1.99)	0.0261	
Older adults	6 (5)	29 (23)	65 (51)	1.70 (0.97–3.02)	0.0671	
**Avoiding face**						
Young adults (ref)	38 (149)	41 (161)	21 (81)	1.0	–	0.127
Middle-aged adults	26 (90)	49 (169)	24 (84)	1.43 (1.06–1.93)	0.0203	
Older adults	35 (28)	38 (30)	27 (21)	1.50 (0.89–2.53)	0.1299	
Working from home^*^						
Young adults (ref)	16 (61)	10 (38)	75 (292)	1.0	–	0.0279
Middle-aged adults	25 (87)	13 (44)	62 (212)	0.45 (0.31–0.64)	<0.0001	
Older adults	51 (40)	4 (3)	46 (36)	0.23 (0.13–0.41)	<0.0001	
**Canceling travel**						
Young adults (ref)	5 (20)	8 (30)	87 (341)	1.0	–	0.7509
Middle-aged adults	7 (23)	12 (40)	82 (280)	0.65 (0.41–1.02)	0.0650	
Older adults	8 (6)	16 (13)	76 (60)	0.66 (0.33–1.34)	0.2393	
**Age group**	**Moderately effective or less**	**Effective**	**Very effective**	**Odds ratio (95% CI)**	***P*****-value**	
**PERCEIVED EFFECTIVENESS OF HEALTH BEHAVIORS**						
**Staying home**						
Young adults (ref)	11 (43)	35 (135)	54 (213)	1.0	–	0.3987
Middle-aged adults	12 (41)	32 (111)	56 (191)	1.09 (0.80–1.49)	0.5728	
Older adults	9 (7)	35 (28)	56 (44)	0.95 (0.56–1.62)	0.8407	
**Social distancing**						
Young adults (ref)	15 (59)	41 (162)	43 (170)	1.0	–	0.4058
Middle-aged adults	9 (30)	38 (129)	54 (184)	1.69 (1.24–2.31)	0.0009	
Older adults	8 (6)	22 (17)	71 (56)	3.33 (1.87–6.07)	0.0001	
**Avoiding crowds**						
Young adults (ref)	5 (21)	27 (107)	67 (263)	1.0	–	0.8018
Middle-aged adults	4 (15)	21 (73)	74 (255)	1.43 (1.00–2.04)	0.0479	
Older adults	3 (2)	11 (9)	86 (68)	2.99 (1.48–6.52)	0.0036	
**Washing hands**						
Young adults (ref)	6 (25)	34 (132)	60 (234)	1.0	–	0.3725
Middle-aged adults	4 (14)	29 (98)	67 (231)	1.39 (1.0–1.94)	0.0512	
Older adults	6 (5)	20 (16)	73 (58)	2.17 (1.2–4.05)	0.0123	
**Avoiding face**						
Young adults (ref)	14 (54)	34 (132)	52 (205)	1.0	–	0.3175
Middle-aged adults	10 (33)	32 (111)	58 (199)	1.30 (0.95–1.79)	0.0968	
Older adults	14 (11)	25 (20)	61 (41)	1.71 (0.99–3.00)	0.0554	
**Working from home**
Young adults (ref)	11 (43)	30 (118)	59 (230)	1.0	–	0.0071
Middle-aged adults	14 (48)	26 (90)	60 (205)	1.10 (0.80–1.52)	0.5518	
Older adults	20 (16)	22 (17)	58 (46)	1.11 (0.65–1.92)	0.7097	
**Canceling travel**						
Young adults (ref)	8 (30)	22 (87)	70 (274)	1.0	–	0.1563
Middle-aged adults	8 (26)	19 (66)	73 (251)	1.30 (0.91–1.86)	0.1473	
Older adults	63 (50)	25 (20)	11 (9)	1.71 (0.92–3.27)	0.0957	

*Young adult as reference, OR = 1.0; Young adults = 18–34 years; Middle-aged adults = 35–64 years; Older adults = 65+ years*.

* *Ethnicity control variable dropped due to convergence issues*.

Across age groups, both middle-aged and older adults indicated that they believed the health behaviors queried were either similarly effective or more effective compared to young adults.

### Perceptions of Adequacy of Health Behaviors Across Age Groups

There were no significant differences across age groups in perceived adequacy of youths' health behaviors to prevent COVID-19 transmission (middle-aged vs. young adults: OR 0.99, 95% CI 0.69–1.41; older vs. young adults: OR 0.96, 95% CI 0.51–1.77). It should be noted that the control variable for education was dropped due to convergence issues for this model. There were differences for perceived adequacy of health behaviors across all adult age groups. Middle-aged adults were less likely to believe that young adults were doing enough to prevent COVID-19 transmission compared to young adults themselves (OR 0.53, 95% CI 0.37–0.75). It should be noted that control variable for urban living was dropped due to convergence issues for this model. Both middle-aged and older-adults were more likely to perceive their own age groups (OR 2.88, 95% CI 2.08–3.99 and OR 2.35, 95% CI 1.73–3.20, respectively) and each other's age groups (OR 2.88, 95% CI 1.65–5.04 and OR 2.35, 95% CI 1.73–3.20, respectively) as responding adequately compared to young adults.

### Model of COVID-19 Health Beliefs and Behaviors

Overall, the structural equation model had a RMSEA of 0.07, a CFI of 0.91, and a SRMR of 0.05. The SRMR value indicates a good fit. On the other hand, the RMSEA and CFI do not meet the Hu and Bentler (1999) definition of “good” fit but can be considered an “acceptable” fit (Brown, 2015). Taken together, it appears that our model provides “acceptable” fit to the data and interpretation of the model is appropriate.

**Figure 1:**
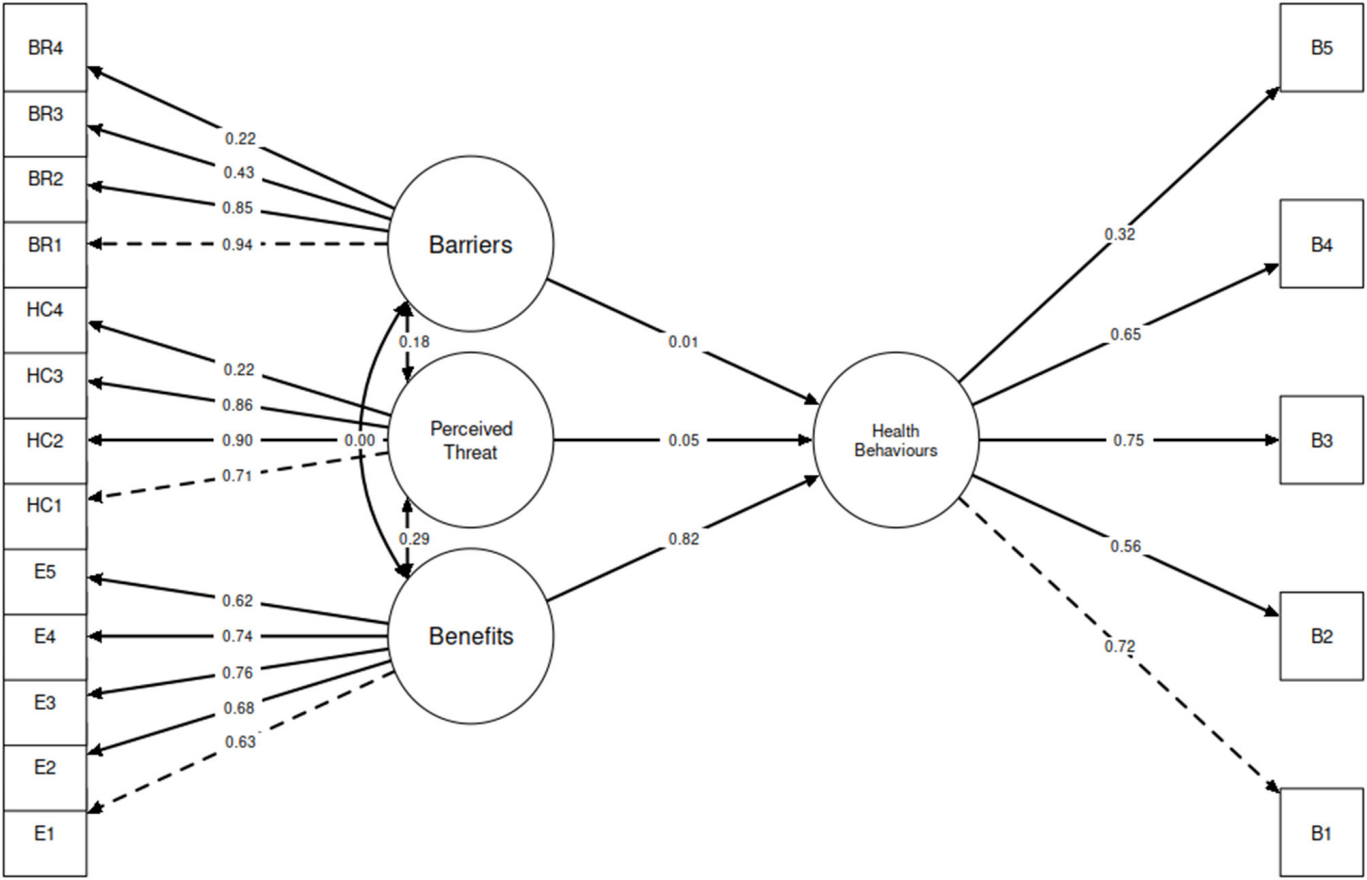
Structural equation modelling describing the relationship between perceived severity and susceptibility of COVID-19, barriers, perceived effectiveness of health behaviors, and socioeconomic variables on the uptake recommended health behaviors (all coefficients are standardized).

The structural equation model diagram is presented in Figure 1, with all coefficients standardized. All factor loadings were significant at the 0.001 level. The only significant predictor of the adoption of health behaviors was the perceived benefits of health behaviors (standardized coefficient: 0.82, *p* < 0.001). Socioeconomic barriers (standardized coefficient: 0.01, *p* = 0.772) and perceived severity and susceptibility (standardized coefficient: 0.05, *p* = 0.132) were not significant predictors of health behavior adoption. Though not directly affecting health behavior adoption, the perceived severity and susceptibility latent variable was directly correlated with the perceived benefits of health behaviors (standardized coefficient: 0.29, *p* < 0.001). Socioeconomic barriers were not directly correlated with perceived benefits of health behaviors (standardized coefficient: 0.00, *p* = 0.001), though they were directly correlated with perceived severity and susceptibility (standardized coefficient: 0.18, *p* < 0.001).

## Discussion

This study surveyed adults across young to older age groups to understand their beliefs and behaviors related to COVID-19 and perceptions of other age groups' responses to the COVID-19 pandemic. Our study yielded several findings that contribute to the evolving literature on health beliefs and behaviors surrounding COVID-19. Prior reports have not investigated the perceptions of COVID-19 impacts, health beliefs, and health behaviors of one's own age group as well as other age groups. Our results suggest that different age groups have distinct perceptions of the health, social, and economic impacts of COVID-19 for people of their own and other age groups. Despite differing perceptions of risk and impact, adoption and perceived effectiveness was largely similar across age groups with high perceived efficacy and high levels of adoption. However, there are some significant differences between age groups related to occupational, personal health, and social behaviors. As well, despite similar adoption of health behaviors across age groups, middle-aged and older adults both perceived members of their own and each others' age groups as more adequate to reduce the spread of COVID-19 compared to how they perceived the behaviors of young adults. In modeling the interaction between perceived benefits, perceived severity and susceptibility, and behavior using the HBM, our findings suggest belief in the benefits of health behaviors are the most important factor driving their adoption amongst all age groups. Our model also suggests perceived severity and susceptibility of COVID-19 to oneself and socioeconomic factors may indirectly affect adoption by altering perceived efficacy of the behaviors.

The perceived impacts of COVID-19 on the health, economic, and social well-being of oneself and others vary by age group in mostly expected ways. In the present study, both middle-aged and older adult respondents were more concerned about the risk of COVID-19 hospitalization and death than younger adult respondents but were not more concerned about COVID-19 infection despite increased susceptibility. This aligns with research showing an age-related increase in COVID-19 severity (Guo et al., 2020), as well as with prior research from a sample of American older adults (Bruine de Bruin, 2020) that found older age was not associated with higher perceived risk of getting COVID-19 but was associated with greater perceived risk of dying due to COVID-19 (Bruine de Bruin, 2020). Another study using data from an American adult sample also found adults of all ages tend to underestimate their risk of being infected with and dying due to COVID-19 (Niepel et al., 2020). Other research has had variable findings about the association between age and COVID-19 risk perception. A Turkish survey of health beliefs found age was negatively associated with perceived vulnerability, risk of, and fear of COVID-19 (Yildirim et al., 2020). Another survey found that older American men were less worried about COVID-19 than younger adults despite perceiving higher COVID-19 risk for themselves (Barber and Kim, 2020). While associations between age and different types of COVID-19 risk (e.g., infection, hospitalization, death) vary in the literature, findings from the present study considered in light of prior research confirm that concerns about the personal health impacts of COVID-19 vary with age.

The Socio-emotional Selectivity Theory (SST) proposed by Carstensen (1995) may provide some explanation into the discrepancies between the perceived susceptibility vs. the risk of death among middle-aged and older adults in our study. SST suggests that as one ages, most of the attention is focused on present-moment events, placing major emphasis on finding meaning and positive emotion, and less focus on future circumstances (Carstensen, 1995). As such, older adults and, to a lesser extent, middle-aged adults, may have a decreased perception of susceptibility, while acknowledging their increased risk of death. This aligns with an Italian study in which older adults showed a more positive outlook and attitude toward the COVID-19 pandemic compared to other age groups (Ceccato et al., 2020). In another study, older adults reported fewer negative emotions related to exposure to COVID-19, despite still reporting an increased perceived risk when compared to other age groups (Carstensen et al., 2020). These findings suggest that older adults may indeed focus more on positive and less on negative aspects of the COVID-19 pandemic, aligning with the SST.

Our results suggest middle-aged adults have greater levels of concern about the impacts of COVID-19 on health, economic, and social well-being across age groups, while there were fewer differences between the beliefs of older and young adults. Middle-aged respondents were more concerned about the impacts of COVID-19 to both their own health and their ability to meet financial obligations during COVID-19 compared to young adults, whereas older adults were no more concerned than young adults. Only for impacts to maintaining social connections and economic futures were older adults also more concerned than young adults. The reason for peak concern among middle-aged adults is unclear. A possible explanation is that middle-aged adults are more likely to have dual care responsibilities, still caring for their children while also caring for aging parents. The combination of caregiving responsibilities with regular family stressors may be exacerbated in middle-aged adults when considering the possible impacts of COVID-19 on the family unit (Prime et al., 2020). It is also possible that the higher concerns amongst middle-aged adults could be related to their specific age cohort. However, the “middle-aged” group as defined in this study spans several generational cohorts, including elder millennials (individuals born in 1980–1990's), Generation X (individuals born 1965–1979), and the youngest Baby Boomers (individuals born 1964–1946). Further research should specifically investigate the impacts of COVID-19 on middle-aged adults' mental well-being, as well as physical, economic, and social well-being, as our research suggests they have heightened concerns compared to older and younger adults.

Respondents in our study reported high uptake of health behaviors, with few differences across age groups. A Turkish study also found high adherence to health behaviors, with the sample reporting increases in all health behaviors assessed due to the COVID-19 pandemic, although it did not report frequency of health behaviors (Yildirim et al., 2020). In the present study, the only significant difference in engagement in health behaviors between age groups was for working from home. This contrasts with findings of a survey of Turkish adults that found younger age was associated with higher frequency of self-reported health behaviors for preventing COVID-19 infection (Yildirim et al., 2020). It is possible that adoption of health behaviors in this Canadian sample was driven by intrinsic factors (e.g., perceptions and beliefs) rather than extrinsic/situational factors (e.g., employment) as respondents reported similar perceived effectiveness of most health behaviors across age groups (with the exception of social distancing and avoiding crowds).

Confirming the importance of intrinsic beliefs, the structural equation model indicated that perceived benefits were the major (and only significant, direct) driver of uptake of COVID-19 health behaviors. This finding aligns with a prior study that used the HBM to assess uptake of a COVID-19 tracing app in a German sample (Walrave et al., 2020). This study also found that perceived benefits of using the app was a greater driver of use of a COVID-19 tracing app than perceived COVID-19 risk. In a study of HBM constructs and uptake of health behaviors in a Chinese sample, perceived benefits of behaviors and rewards for engaging in behaviors were positively associated with practicing good hand hygiene, wearing a face mask, and social distancing (Tong et al., 2020). Studies have also used different theories to understand health behaviors under COVID-19. The Extended Parallel Process Model is similar to HBM and describes behavior as a result of perceived threat (severity and susceptibility) of the outcome and self-efficacy for reducing the threat (Lithopoulos et al., 2021), with additional moderators. This model was used as a framework in recent study of Canadian adults where both perceived threat and self-efficacy for behaviors predicted uptake of health behaviors. However, self-efficacy for reducing the threat (belief that their behaviors will be beneficial) was most strongly related to behavior uptake (Kim and Crimmins, 2020a). Protection Motivation Theory (PMT) (Rogers, 1975) also includes perceived threat (severity and susceptibility) as a predictor of behavior and intentions as well as coping resources (efficacy of behavior and self-efficacy for the behavior). One study using PMT as a framework surveyed people in Wave 1 (March 2020) and Wave 2 (May 2020) (Rogers, 1975). Results indicated that older adults and women were more likely to report that protective behaviors were effective in reducing COVID-19 spread and were more likely to adopt these behaviors (Kowalski and Black, 2021). Another study compared behaviors between younger and older adults (Kim and Crimmins, 2020a). Similar to the current study, perceived coping resources (including perceived efficacy of the behavior) predicted behavior uptake among young adults. However, in contrast to the current study, behavior of older adults was more strongly predicted by perception of COVID-19 severity (Kim and Crimmins, 2020a). Other research (without a guiding framework), also found increased perceived risk of being infected with or dying from COVID-19 was associated with higher uptake of health behaviors (e.g., increased hand washing, avoiding crowds) in an American sample (Niepel et al., 2020). A survey from Turkey also found that fear of COVID-19 infection, perceived risk, and vulnerability were significant predictors of engagement in recommended health behaviors (Yildirim et al., 2020). However, neither of these latter studies assessed beliefs about the benefits of health behaviors, so it is unclear the relative importance of perceived benefits and risks in these studies.

The finding that benefits are the primary driver of behavior uptake has important implications for both designing public health communications and for interpreting why some health behaviors are not as well-adopted. In particular, the uptake of masks in Canada, which was not captured in this study as it was not a recommended health behavior for COVID-19 prevention at the time of data collection, is more controversial than other health behaviors. This may be caused by variable messaging around the benefits of masks, where early public health communications stated that there were few if any benefits of masks, and global mask-wearing recommendations have been multifarious (Feng et al., 2020). While these messages have been updated, our study suggests that the inconsistent messaging regarding the benefits of masks may be a reason for poor uptake, rather than the perceived barriers to uptake (e.g., discomfort, cost). Public health messaging may be more effective at increasing the uptake of health behaviors if it highlights the benefits of health behaviors to prevent COVID-19, rather than focusing solely on communicating risk related to COVID-19.

Our study found differences in the perceptions of age groups about the adequacy of other age groups' health behaviors in responding to the COVID-19 pandemic. Specifically, each age group rated their own age group more highly than others age groups did. This finding may be the result of cognitive biases that cause one to perceive their own abilities as superior to others. The egocentric “better than average” or Illusory Superiority bias proposes that people tend to assume their own traits or abilities are better than the average (Zell et al., 2020). In the context of COVID-19 health behaviors, egocentric bias could lead to one assuming that one is doing more than others to prevent COVID-19 transmission. Combined with group attribution error (Mackie and Allison, 1987) and ingroup bias (Taylor and Doria, 1981), this may lead one to assume that one's own age group is doing more than others to prevent COVID-19 transmission. Group attribution error posits that individuals assume that all members of a group share the same traits, and ingroup bias posits that individuals view others more positively when they have shared characteristics, which may further lead to people rating their own age group more highly.

Our study also found that younger adults' behaviors were more poorly perceived by older age groups, with middle-aged and older adults more likely to perceive their own and each other's health behaviors as adequate to reduce COVID-19 transmission compared to younger adults. News and social media reports documenting specific instances where health behaviors were not adhered to by specific age groups early in the COVID-19 pandemic (e.g., young adults participating in large social gatherings with lack of social distancing during spring break and St. Patrick's Day festivities) could have led to the early formation of biased perceptions amongst other age groups. Group attribution error may then cause this to become a stereotypical belief about poor COVID-19 health behaviors among younger adults as a whole. While younger adults were less likely to report belief in the efficacy of some health behaviors and adhering to social and occupational recommendations to prevent COVID-19 than middle-aged and older adults, it's important to note that reported levels of adherence were high across all age groups. With the exception of avoiding touching one's face, more than 80% of younger adults who responded reported engaging in all health behaviors queried “very often” or “always.”

This study has limitations that must be considered when interpreting these findings and their applicability to other populations. First, the participants of our study are not representative of the broader population; ours is a highly educated, predominantly white, and largely female sample residing in urban areas in Ontario, Canada. This is likely due in part to the use of a convenience sampling approach to survey recruitment conducted over social media, but there is also a systemic trend in research for participation to be biased toward people with higher socioeconomic status and Caucasian ethnicity (Oh et al., 2015). COVID-19 has had vastly different effects in different countries, and disproportionately affects ethnic minority groups and people with lower socioeconomic status (Hawkins et al., 2020; Sze et al., 2020). As such, findings from this study are not representative of the population as a whole or groups that are most vulnerable to COVID-19. In addition, the survey was cross-sectional. As a result, these findings depict the health beliefs and behaviors of a specific subset of the population at a single time, which may or may not be reflected by other socioeconomic or ethnic groups or changing mindset over the pandemic. Further research should seek to explore the health beliefs and behaviors of more vulnerable socioeconomic groups to understand how this may affect uptake of COVID-19 health behaviors, as a significant but indirect association of socioeconomic factors was associated with health behavior uptake in our study.

These results reflect a snapshot of COVID-19 health beliefs and behaviors, which will continue to evolve as we live with the global COVID-19 pandemic. Data collection occurred from May to June 2020, which was relatively early in the pandemic, and thus does not necessarily reflect how health behavior adherence evolves over time. One study from a sample representative of the American population found older adults were more likely to sustain uptake of health behaviors for preventing COVID-19 transmission, but reduction in “risky” health behaviors did not vary as the pandemic progressed (Hawkins et al., 2020). These results suggest that ongoing assessment of health perceptions and behaviors in the COVID-19 pandemic, including those related to vaccine efficacy, is important to tailoring health behavior interventions and messaging.

Though the perceived impact of COVID-19 varied across age groups in predictable ways, our study indicates that adoption of health behaviors to contain COVID-19 is high with few differences across age groups, at least among our limited sample. Of specific importance for public health communications, the perceived benefits of the health behaviors, and not the risk of COVID-19, appeared to be the only significant, direct driver of adoption. Public health communications regarding health behaviors should be designed with this in mind. Future research should probe the impact of COVID-19 on middle-aged adults more deeply, as this age group was most concerned about the impact of COVID-19 among all age groups.

## Data Availability Statement

The original contributions presented in the study are publicly available. This data can be found here: https://osf.io/q28mh/.

## Ethics Statement

This study was reviewed and approved through a University of Waterloo Research Ethics Committee. Written informed consent for participation was not required for this study in accordance with the national legislation and the institutional requirements.

## Author Contributions

LB, MB, BN, TD, and LM contributed to the research concept development, research design, data collection, data analysis, interpretation, and manuscript preparation for this research study. All authors contributed to the article and approved the submitted version.

## Conflict of Interest

The authors declare that the research was conducted in the absence of any commercial or financial relationships that could be construed as a potential conflict of interest.

## Footnotes

^1^https://osf.io/q28mh/
