# Supplementary material for: Using the Health Belief Model to Understand Age Differences in Perceptions and Responses to the COVID-19 Pandemic
Source: Front Psychol. 2021 Apr 15;12:609893. doi: 10.3389/fpsyg.2021.609893 (PMC8082183; doi:10.3389/fpsyg.2021.609893)
Supplement: Supplementary file 1 [file Data_Sheet_1.DOCX]

**Supplementary Information: Survey Questions**

**Sociodemographic Information**

1. Please indicate your age as of today, in years.
2. Please indicate your gender:
   - Man
   - Woman
   - Non-binary
   - Prefer to self-identify (option to specify): ___________
3. Have you had a close family member or friend who has, or has, had COVID-19?
   - Yes
   - No
4. Have you tested positive for COVID-19?
   - Yes
   - No
   - [If no to Question 3] Do you suspect that you have, or have had, COVID-19?
     - Yes
     - No
   - [If no to Question 3] Do you suspect you have been exposed for COVID-19?
     - Yes
     - No

**COVID-19: Impacts on me**

1. How concerned are you about each of the following impacts of COVID-19 for your PERSONAL HEALTH?

|  | Not at all concerned | Somewhat concerned | Moderately concerned | Very concerned | Extremely concerned |
| --- | --- | --- | --- | --- | --- |
| Being infected myself |  |  |  |  |  |
| Being hospitalized if you were infected |  |  |  |  |  |
| Dying of COVID-19 if you were infected |  |  |  |  |  |
| Other, please specify: |  |  |  |  |  |

1. How concerned are you about each of the following each of the impacts of COVID-19 for your SOCIAL AND FINANCIAL WELL-BEING?

|  | Not at all concerned | Somewhat concerned | Moderately concerned | Very concerned | Extremely concerned |
| --- | --- | --- | --- | --- | --- |
| Maintaining social connections |  |  |  |  |  |
| My economic future |  |  |  |  |  |
| Ability to meet financial obligations |  |  |  |  |  |
| Other, please specify: |  |  |  |  |  |

1. How **often** do you employ the following precautions to reduce your risk of exposure to COVID-19?

|  | Never | Rarely | Sometimes | Very often | Always |
| --- | --- | --- | --- | --- | --- |
| Avoiding leaving the house for non-essential reasons |  |  |  |  |  |
| Using social distancing when out in public |  |  |  |  |  |
| Avoiding crowds and large gatherings |  |  |  |  |  |
| Washing your hands more frequently |  |  |  |  |  |
| Avoiding touching your face |  |  |  |  |  |
| Working from home |  |  |  |  |  |
| Cancelling non-essential travel |  |  |  |  |  |
| Other, please specify: |  |  |  |  |  |

1. How **effective** do you think the following precautions are at reducing your risk of exposure to COVID-19?

|  | Never | Rarely | Sometimes | Very often | Always |
| --- | --- | --- | --- | --- | --- |
| Avoiding leaving the house for non-essential reasons |  |  |  |  |  |
| Using social distancing when out in public |  |  |  |  |  |
| Avoiding crowds and large gatherings |  |  |  |  |  |
| Washing your hands more frequently |  |  |  |  |  |
| Avoiding touching your face |  |  |  |  |  |
| Working from home |  |  |  |  |  |
| Cancelling non-essential travel |  |  |  |  |  |
| Other, please specify: |  |  |  |  |  |

**COVID-19: Impacts on others**

1. How concerned are you about the impacts of COVID-19 on the **HEALTH** of:

|  | Not at all concerned | Slightly concerned | Moderately concerned | Very concerned | Extremely concerned |
| --- | --- | --- | --- | --- | --- |
| Youth? (under 18 years) |  |  |  |  |  |
| Young adults? (18-39 years) |  |  |  |  |  |
| Middle-aged adults? (40-64 years) |  |  |  |  |  |
| Older adults? (65 years and older) |  |  |  |  |  |

1. How concerned are you about the impacts of COVID-19 on the **ECONOMIC FUTURE** of:

|  | Not at all concerned | Slightly concerned | Moderately concerned | Very concerned | Extremely concerned |
| --- | --- | --- | --- | --- | --- |
| Youth? (under 18 years) |  |  |  |  |  |
| Young adults? (18-39 years) |  |  |  |  |  |
| Middle-aged adults? (40-64 years) |  |  |  |  |  |
| Older adults? (65 years and older) |  |  |  |  |  |

1. How concerned are you about the impacts of COVID-19 on the **SOCIAL WELL-BEING** of:

|  | Not at all concerned | Slightly concerned | Moderately concerned | Very concerned | Extremely concerned |
| --- | --- | --- | --- | --- | --- |
| Youth? (under 18 years) |  |  |  |  |  |
| Young adults? (18-39 years) |  |  |  |  |  |
| Middle-aged adults? (40-64 years) |  |  |  |  |  |
| Older adults? (65 years and older) |  |  |  |  |  |

1. Do you think the following age groups are doing enough to reduce the spread of COVID-19?

|  | Definitely not | Probably not | Might or might not be | Probably yes | Definitely yes |
| --- | --- | --- | --- | --- | --- |
| Youth (under 18 years) |  |  |  |  |  |
| Young adults (18-39 years) |  |  |  |  |  |
| Middle aged adults (40-64 years) |  |  |  |  |  |
| Older adults (65 years and older) |  |  |  |  |  |

**Additional demographics**

1. What is your current employment status?
   - Employed
   - Self-employed
   - Not employed, but looking
   - Not employed, by choice
   - Not employed, not able to work
   - Retired
2. Are you a student?
   - Yes
   - No
3. Is your occupation health-related?
   - Yes
   - No
   - [If yes to Question 15] If your occupation is health related, please specify what kind of role:
     - Healthcare provider (includes physicians, nurses, allied health professionals, and personal support workers)
     - Healthcare administrator
     - Public health professional
     - Community health worker
     - Health researcher
     - Other (please specify):
4. What is your highest level of education?
   - Less than high school
   - High school diploma, or equivalent
   - Some post-secondary education (university, college, or trade school)
   - Postsecondary certificate, diploma, or degree
   - Post-graduate degree
5. In a typical year, what is your approximate annual household income?
   - Less than $20,000 CAD
   - $20,000 CAD - $50,000 CAD
   - $50,000 CAD - $100,000 CAD
   - $100,000 CAD - $150,000 CAD
   - $150,000 CAD or more
6. What is your ethnic background? (dSelect all that apply)
   - First Nation or North American Indian
   - Alaskan Native
   - Métis
   - Other Aboriginal or Indigenous
   - European origins (Caucasian)
   - European origins (not Caucasian)
   - Black / African Canadian / African American
   - Caribbean origins
   - Latin, Central, and South American origins
   - African origins
   - West Central Asian and Middle Eastern origins (e.g. Turkish, Iranian)
   - South Asian origins
   - Oceania originas (e.g. Hawaiian, Samoan)
   - Prefer not to answer
7. If your ethnic background is not reflected in the response options above, please specify
8. What is your country of residence? [drop down menu]
9. What is your province or state of residence?
10. What is your town or city of residence?
11. Do you need care or support to help manage your everyday activities?
    - Yes
    - No
12. On a scale of 1 to 100, with 0 being “poor” and 100 being “excellent”, how would you rate your:
    - Physical Health [sliding integer scale 0-100]
    - Mental health [sliding integer scale 0-100]
